# Automated detection and recognition system for chewable food items using advanced deep learning models

**DOI:** 10.1038/s41598-024-57077-z

**Published:** 2024-03-19

**Authors:** Yogesh Kumar, Apeksha Koul, Kamini, Marcin Woźniak, Jana Shafi, Muhammad Fazal Ijaz

**Affiliations:** 1 Department of CSE, School of Technology, Pandit Deendayal Energy University, Gandhinagar, Gujarat India; 2 Department of Computer Science and Engineering, Punjabi University, Patiala, Punjab India; 3 Southern Alberta Institute of Technology, Calgary, Alberta Canada; 4 Faculty of Applied Mathematics, Silesian University of Technology, Kaszubska 23, 44100 Gliwice, Poland; 5 Department of Computer Engineering and Information, College of Engineering in Wadi Al Dawasir, Prince Sattam Bin Abdulaziz University, 11991 Wadi Al Dawasir, Saudi Arabia; 6 School of IT and Engineering, Melbourne Institute of Technology, Melbourne, 3000 Australia

**Keywords:** Food identification, Deep learning, Eating sounds, Customized convolutional neural networks, Audio signal processing, Spectrograms, Mel-frequency cepstral coefficients, Computational science, Computer science, Information technology, Software

## Abstract

Identifying and recognizing the food on the basis of its eating sounds is a challenging task, as it plays an important role in avoiding allergic foods, providing dietary preferences to people who are restricted to a particular diet, showcasing its cultural significance, etc. In this research paper, the aim is to design a novel methodology that helps to identify food items by analyzing their eating sounds using various deep learning models. To achieve this objective, a system has been proposed that extracts meaningful features from food-eating sounds with the help of signal processing techniques and deep learning models for classifying them into their respective food classes. Initially, 1200 audio files for 20 food items labeled have been collected and visualized to find relationships between the sound files of different food items. Later, to extract meaningful features, various techniques such as spectrograms, spectral rolloff, spectral bandwidth, and mel-frequency cepstral coefficients are used for the cleaning of audio files as well as to capture the unique characteristics of different food items. In the next phase, various deep learning models like GRU, LSTM, InceptionResNetV2, and the customized CNN model have been trained to learn spectral and temporal patterns in audio signals. Besides this, the models have also been hybridized i.e. Bidirectional LSTM + GRU and RNN + Bidirectional LSTM, and RNN + Bidirectional GRU to analyze their performance for the same labeled data in order to associate particular patterns of sound with their corresponding class of food item. During evaluation, the highest accuracy, precision,F1 score, and recall have been obtained by GRU with 99.28%, Bidirectional LSTM + GRU with 97.7% as well as 97.3%, and RNN + Bidirectional LSTM with 97.45%, respectively. The results of this study demonstrate that deep learning models have the potential to precisely identify foods on the basis of their sound by computing the best outcomes.

## Introduction

The crunch of their products is now being commercialized by some of the biggest food companies in the world. When a product's acoustic qualities, such as crispy, crunchy, crackly, etc., are crucial, marketers will often emphasize these attributes in TV commercials to highlight how important sound is to a product's overall appeal. The marketed product's flavour or scent cannot be experienced by viewers of television. They are only able to see it and, naturally, hear it. Advertising a food product's audio features introduces potential customers to this crucial quality trait of many goods^1^. Crunchiness, hardness, and crispness, to name just a few, are texture-related attributes that affect a product's appeal. Thanks to recent advancements in analysis technology, such as the Acoustic Envelope Detector attached to a TA.XTplus Texture Analyzer, manufacturers are now able to extract this valuable data. Once product designers have created a popular "noisy" food product, the goal is to maintain this distinctive quality of the product throughout manufacturing. Every brand is aware of the significance of consistent product quality, whether it is in flavour, appearance, or texture. In order to establish the benchmark product "noise" for quality control of all ensuing batches of the product, it is crucial to measure a product's acoustic signature^2^.

In order to create a louder product, you would seek out the one that produces the largest "peaks" or decibel values, i.e., towering peaks as opposed to numerous small ones. The number of peaks produced can be counted, and the number of seconds over which they occur divided, to compare the crispiness of various items. You may find out from this how many fractures are made every second; the more, the crispier the result. These sounds impart knowledge to the prospective customer. The listener or observer can first gauge the degree or severity of crunchiness and sharpness^3^. They ascertain this by taking note of the overall volume of sound generated at a specific biting distance. The presence of a sizable percentage of high-pitched noises denotes crispiness in the product. The result is crunchier if lower-pitched noises make up a larger percentage of the sound spectrum. Due to the brittle breakage of the cell walls, crunchy food produces distinct sounds when broken or crushed. Cracks spread at speeds that are too fast for even high-speed camera; therefore the sound is created in a brief period of time, or as a pulse^4^. The pulses, which appear as a succession of tall peaks when slowed down and plotted onto a graph, only persist for a few milliseconds. Simply said, the crispier it is, the more peaks there are. Acoustic emission has been used to measure the sharpness of the senses. The loudness of the sounds from crisp foods sets them apart from non-crisp dishes. Louder noises would be produced by crisper products since amplitude is a factor that separates more crisp sounds from ones that are less sharp^5^.

In this article, we propose the deep learning models which include LSTM, GRU, Hybrid of RNN and Bidirectional LSTM, Hybrid of RNN and GRU, Hybrid of Bidirectional LSTM and GRU and InceptionResNetV2 for the identification of different types of food sounds which may benefits to the food and media industries. Initially, data pre-processing and exploratory data analysis of the eating sounds is performed with libraries such as Tensorflow, Seaborn. The primary idea is to use the eating food sounds of 20 categories of the foods by loading audio sound files and then apply feature extraction techniques which include spectrogram for visually representing the strength of the signal. The spectral rolloff is further used to measure the shape of the signal for computing the rolloff frequency for each frame. After that, spectral bandwidth represents the lower and upper frequencies in a continuous band of frequencies. Then further, MFCCs captured the timbral and textural aspects of sound. For extracting MFCCs, a Fourier Transform is applied to move from the time domain to the frequency domain for extracting the frequency domain features. Then finally, different deep learning techniques are applied to obtain the accuracy: 98.27% for hybrid (Bidirectional LSTM + GRU), 97.48% for Hybrid (Simple RNN + Bidirectional GRU), 97.83% for Hybrid (Simple RNN + Bidirectional LSTM), 94.56% for InceptionResNetV2, 95.57% for LSTM and 99.28% for GRU for eating food sounds identification. Additionally, a CNN model has been proposed, and its parameters have been fine-tuned in such a way that it computes an accuracy of 95.96% for the same dataset.

The remaining structure of the paper includes the contribution of researchers in identifying and classifying various food items using various learning models along with the limitations in Section II. Section III covers a detailed description of the framework used to identify and recognize various items of food on the basis of audio signals. Section IV displays the results in detail, and finally, the complete paper is discussed in section V and is concluded in section VI where the challenges and future scope are mentioned.

## Related work

Recognizing food automatically on the basis of the eating sound is a difficult task but researchers have contributed a lot in this field because the traditional methods did not prove to be successful in order to achieve the best accuracy of classifying food items. But on the other hand, deep learning based techniques have showcased the promising results to identify various food items.

Khan et al. (2022)^6^ had discussed about a novel system i.e. iHearken which is a hardware wearable device in the form of a headphones embedded with sensors in it. This system had been developed for monitoring the eating activity so that the food item could be identified in a real world. The hardware had been designed in such a way which capture data of 16 persons for 20 various food items. The analysis had been done sound of chewing which were later pre-processed with the help of a Finite Impulse Response (FIR) filter and later extracted bottleneck features. Bi-directional long short term memory and softmax function had been used for the calculation the identification score of chewing sound to classify the category of data i.e. whether solid or liquid food category. Likewise, Kojima et al. (2016)^7^ designed a knife device, known as “CogKnife” for the identification of various items of food like apple, banana, leeks, cabbages, and peppers. The knife had been attached with a mini microphone which captured the sound which had been produced during the chopping process. The features had been extracted using the technique spectrogram and were used to train the classifiers such as support vector machine, KNN, and convolutional neural network in the form of feature vectors. Transfer learning based model had been developed by Vijyakumari et al. (2022)^8^ for the classification of 101 different food products in their respective classes. Transfer learning model such as EfficientNetB0 had been trained with the dataset and it computed the accuracy of 80% which proved that the model worked well in terms of its accuracy as compared to their existing techniques. Bluetooth headsets were used by Gao et al. (2016)^9^ for detecting the eating events of the user by analyzing the sound pattern of their chewing any food. The model like support vector machine with conventional kernel based technique were used for the classification and while implementing, the model computed an accuracy of 95% for the tested images but on the contrary, the performance of the system dropped by 65–76% when applied on real world data. Hence, researchers also worked on deep learning model to overcome the said error and the promising results were shown as the detection accuracy was increased by 77–94% that too in the presence of ambient noise. Uchiyama et al. (2021)^10^ had mentioned about the audio visual model that could generate real food texture on the basis of the visuals of the people eating food without any sound. A magnitude spectrogram had been produced to match the visual information and to generate it from the raw audio audio waves via inverse short –time Fourier transform had been the complex task. Hence to overcome it, the researchers applied Griffin-Lim method for recovering the information from the predicted magnitude spectrogram. A method was proposed by Päßler and Fischer (2014)^11^ to analyze the intake of food type by recording its chewing sound via microphone which had been placed in the outer ear canal. The researchers worked on eight different models which had been designed to automatically detect chewing sounds. The models were examined on the basis of 68,904 chewing sounds and two datasets in which the first dataset included the sound recordings of six type of food that had been taken and second dataset comprised of various environment sounds. While training and testing the model, it had been found that the most of the models computed recall and precision which exceed by 80%. To cancel the noise and improve the quality of the signal, simple noise reduction algorithm had been used along with the spectral subtraction. Amfat et al. (2009)^12^ introduced the prediction of analyzing the chewing sound based on the individual bites to identify the type of food by placing sensor on the ear pad. In their paper, pattern recognition technique had been used for the recognition of eating cycles and identifies the food that had been consumed. The data had been collected from eight participants and was performed for three different food items which had 504 bite weights. Linear models were built to predict the bite weight and classify the food type. The models were evaluated based on their accuracy, recall, and precision which were 94%, 80%, and 70% respectively. During experimentation, it was found that the mean weight prediction error was lowest for apples with 19.4% and highest for lettuce with 31% using the sound-based recognition. In fact, Amfat et al. (2005)^13^ also worked on the automatic dietary monitoring system in which the type of food had been analyzed on the basis of their eating sounds. Microphone had been inserted inside the ear canal to capture the chewing sound of food and during implementation 3500 s of chewing data had been collected from four people who consumed four multiple food items. The model showcased the results up to 99% and achieved classification accuracies ranging from 80 to 100% for identifying different food types.

Food identification technology could help both the food and media industries to easy the people and computers to work together. So, Ma et al. (2020)^14^ used 11,141 YouTube clips of 20 different kinds of food to make a CNN model for classifying food. The grouped holdout evaluation technique was used to test the model, and it was found to be accurate 18.5% of the time. But when the uniform holdout evaluation technique was used, the model was 37.58% more accurate. Also, the model did well for most pairs of food types when the job was looked at as a "binary classification problem." Overall, the method did better than acceptable baseline methods in both settings where it was tested. In fact, data-driven study on eating sounds showed that texture properties and differences in how people eat were very important. Likewise, Rousat et al. (2018)^15^ worked on a way to automatically identify eating behavior from video data. The paper gave an in-depth look at the current state of the art in both active and passive dietary tracking which focused on the problems. The authors also developed a framework for user assistance systems that combined active and passive methods and offered three different levels of help. As part of their methodology, the paper described a proof-of-concept study that used 360-degree camera footage. Also, the suggested framework tried to improve the accuracy and effectiveness of dietary monitoring systems by using both active and passive methods.

Deep neural networks are thought to be good for automatically keeping track of a person's food because they are good at classifying audio events. But they have some problems, like the fact that they are hard to program, waste a lot of energy, and need a lot of memory. To get around these problems, Nyamukuru et al. (2020)^16^ came up with shallow gated recurrent unit (GRU) architecture with limited resources. Researchers made Tiny Eats GRU, a shallow GRU neural network, on an Arm Cortex M0 + low-power microcontroller. During experimentation, it had been found that the Tiny Eats GRU only used 4% of the Arm Cortex M0 + memory and had a lag of 6 ms with a 95.15% accuracy rate when figuring out if an individual was eating or not. Nakamura et al. (2021)^17^ worked on making an automated way to recognize different items of food based on the sounds made while eating. A combined CTC/attention model was used by the researchers to automatically find left chewing, front biting, right chewing, and swallowing. The model was trained with weakly labeled data from sound recordings made with 2-channel microphones placed close to the ear. The researchers used the weakly labeled data to create a bigger set of weakly labeled eating sounds to add to the training data. The performance of recognition was improved by using a model that combined CTC and attention and could learn from its surroundings. Also, the study showed that the model worked well for both open and closed foods. Overall, the method created showed promise for automatically recognizing eating behaviors through sound analysis. This could make healthcare and medical applications easier and more useful. Vasileios et al. (2021)^18^ used an in-ear microphone and developed algorithms which aimed at detecting chewing sounds as well as recognizing three distinct food-texture attributes such as crispiness, wetness (moisture), and chewiness. They used binary Support Vector Machines (SVMs), and proposed two algorithms in which one was used for recognizing each texture attribute at the chew level and another at the chewing-bout level. The researchers evaluated the performance of the algorithm using leave-one-subject-out cross-validation on a dataset which involved 9 subjects. Additionally, leave-one-food-type-out cross-validation was also conducted to analyze the generalization capability of the approach to new, unknown food types. Their results indicated a high level of performance in recognizing crispiness, with a weighted accuracy of 0.95 on new subjects and 0.93 on new food types.

Besides this, a comparison has been also done to compare the work of the researchers in the field to detect and classify food items in Table 1.

**Table 1:** Analysis of the previous work.

Ref	Dataset	Tech	Outcome	Challenges
Khan et al. (2022)^6^	Data of 20 food items	Bi-LSTM, iHearken	Accuracy = 97.42%	The work could be extended by incorporating more advanced techniques for the classification of food items
			Precision = 96.80%	
			Recall = 98%	
			F1 score = 97.51%	
Kojima et al. (2016)^7^	Data of six fruits and vegetables	KNN	Accuracy = 83%	Limited dataset
		SVM	Accuracy = 95%	
		CNN	Accuracy = 89%	
Vijayakumari et al. (2022)^8^	101 different food products	EfficientNetB0	Accuracy = 80%	The model should in future be applied to both image as well as text data
Gao et al. (2016)^9^	Data collected from 28 individuals	SVM	Accuracy = 95%	No diversity had been seen in the dataset
Uchiyama et al. (2021)^10^	Data of food ASMR video collected from YouTube	Spectrogram, inverse STFT, Griffin-Lim algorithm	Perceptual evaluation of speech quality (PESQ) = 1.27	The algorithm could be applied to real time data
Päßler and Fischer (2016)^11^	68,094 chewing sounds	Biomedical signal processing	Precision = 80%	The system needed an optimization to enhance its performance
Amft et al. (2009)^12^	Data taken from eight participants	Pattern Recognition Procedure	Precision = 70%	The model could be applied only for the solid foods
			Recall = 80%	
Amft et al. (2005)^13^	Four various types of food	Hearing aids, Headsets	Accuracy = 99%	Limited dataset

## Methodology

**Figure 1:**
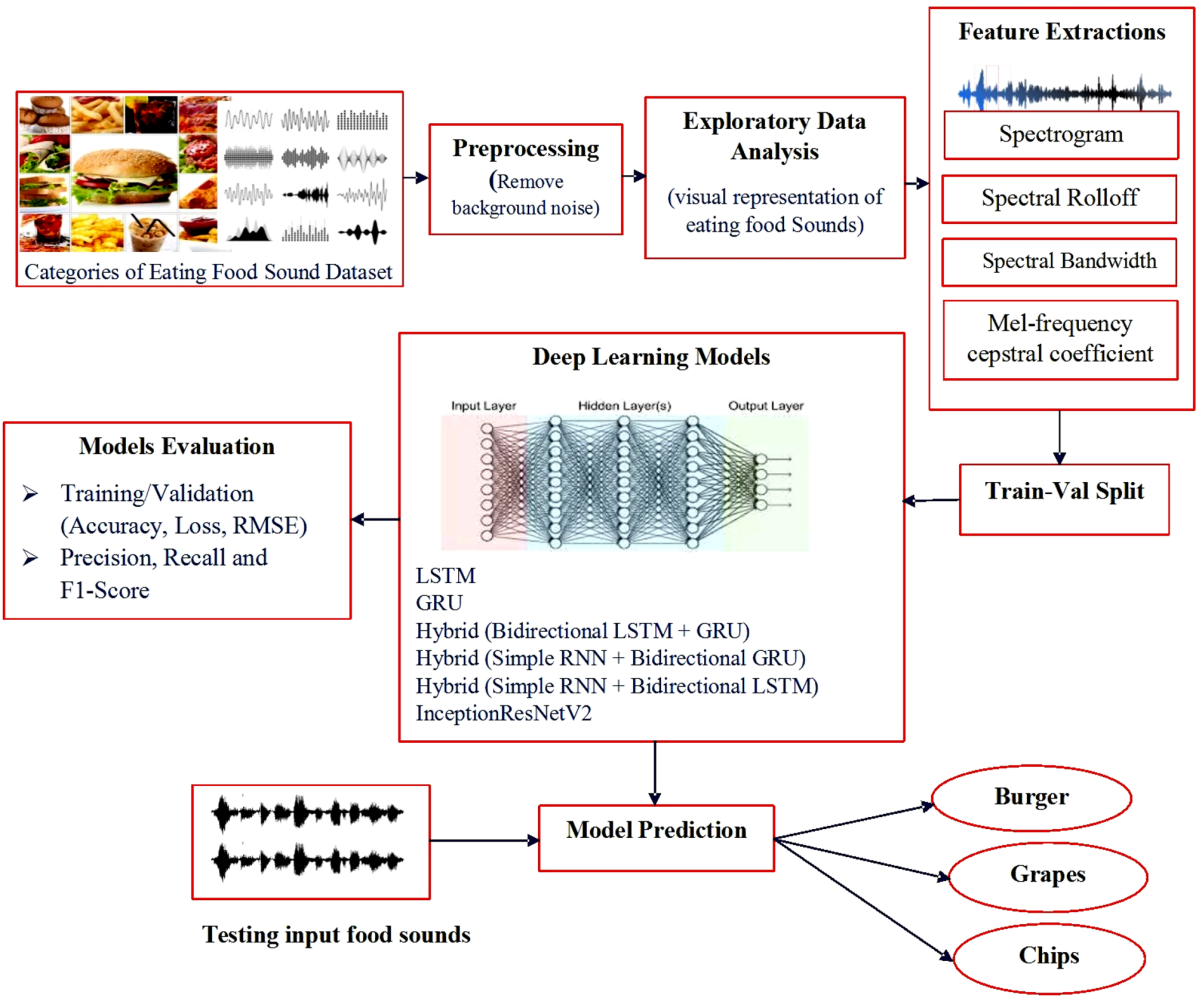
Proposed system of food detection and classification.

This section of the paper presents the framework of the proposed system, as shown in Fig. 1, in which initially the libraries have been used for importing the different food sound datasets having audio files, which are further used for pre-processing, feature extraction, and model performance comparison of applying models for food sound recognition.

### Platforms and libraries used

Several Python libraries, including pandas, librosa, seaborn, matplotlib, sklearn, tensorflow, pathlib, and NumPy, have been used to import the dataset and perform the dataset visualization and cleaning of data to perform food sound feature extractions and classification for eating food prediction. All the used supportive libraries have different purposes for operations to perform the desired task. Likewise, the tensorflow framework is used in the applied deep learning models to perform faster computations for numerical audio data. The computation in tensorflow is described in terms of nodes which perform data movement between nodes such as tensor. Edges define the flow of data, branching and looping in graphs. Where operations in tensorflow take input attributes and produce output attributes to perform different operations such as multiplying, etc.^19^.

### Data descriptions and visualization

The audio data was gathered from publicly available YouTube video sources, with an emphasis on the availability and abundance of content produced by eating-themed channels. The appropriate content based on the top search results for the term 'eating sound,' had been selected and took into account both the popularity of the channels and the variety of food items. This thorough effort resulted in the creation of a food categorization dataset that included 246 YouTube videos encompassing twenty various food classes. Within each class, a thorough selection of 12–14 videos was made which results in to a comprehensive dataset of 11,141 clips each spanning from 1 to 22 s^20^.

In addition, to improve the dataset's quality and relevance, variations in space features, food kinds, recording qualities, and eating behaviours were purposefully introduced during video recording in a controlled room environment.

To extract eating sound samples with precision, Logic Pro X 10.5.1 was used. Notably, the extraction approach focused removing undesired items like chatting, silverware, and packaging sounds. To ensure consistent audio quality across all clips, longer chunks lasting more than 6 s were carefully broken into smaller, more manageable segments. Peak normalization was used to provide uniform audio quality throughout all clip sections, with a target of -1 dB. This normalizing method, which used 0 dB as the distortion edge, helped to retain consistency and improve the overall reliability of the audio data for future study^21^.

**Figure 2:**
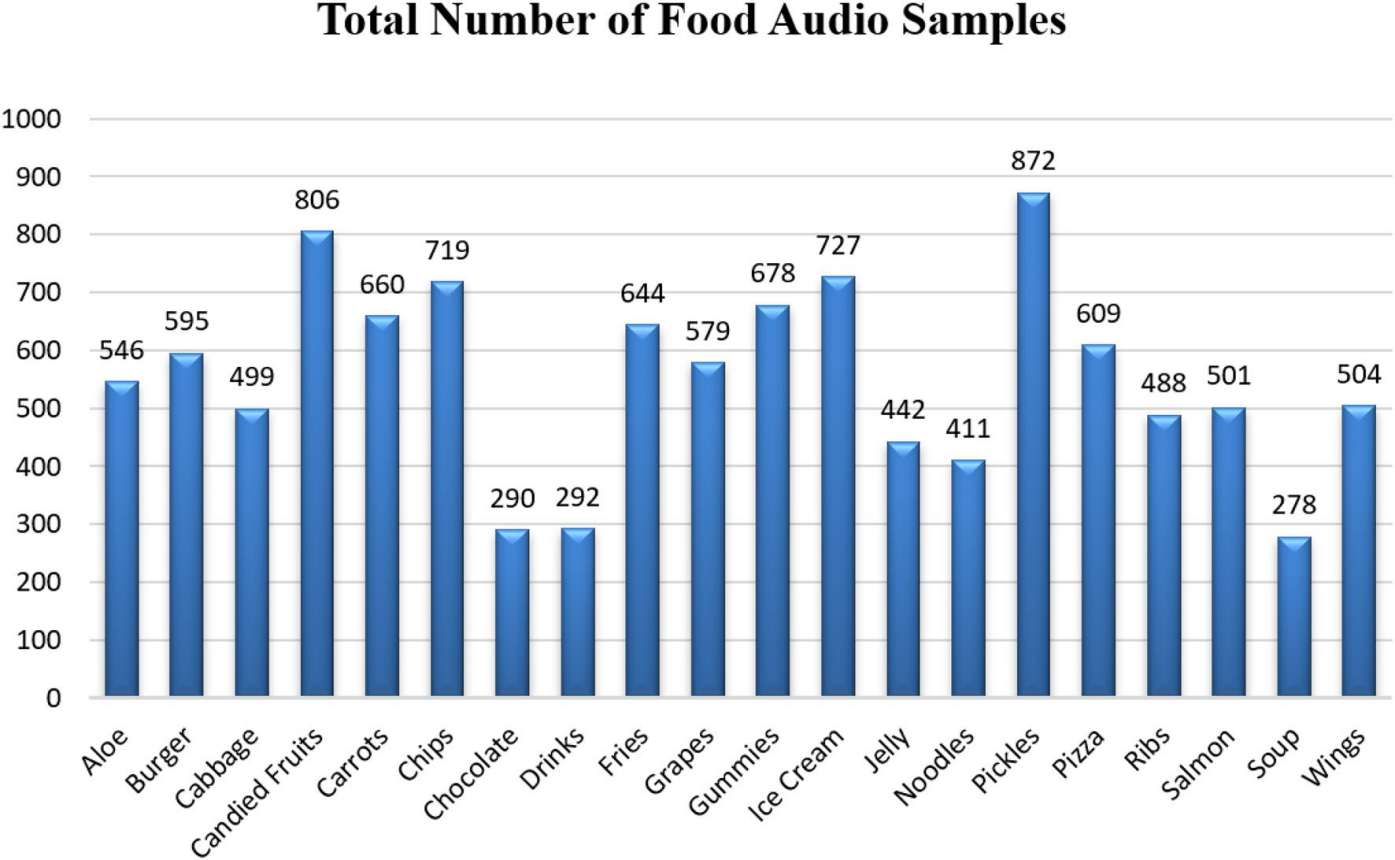
Food categories audio sample count.

For exploratory data analysis, the seaborn python libraries have been used to visualize the different categories of food sounds. Figure 2 also highlights the total number of used food sound clips for each category. The main purpose of exploratory data analysis for food categories is to better understand the patterns within the sound data files to detect outlier or anomalies and to find the relationships between the sound files of different food items. It also helps to manipulate the audio data of eating food items to understand the categorizations.

### Feature extractions

For feature extractions, the spectrograms, spectral rolloff, spectral bandwidth and mel-frequency cepstral coefficients is used for cleaning of audio files for different categories of food. Initially, audio data file is loaded by using librosa library which further visualizing audio file by plotting the audio array using the librosa.display.waveplot class.

#### Spectrogram and spectral rolloff for eating food audio files

A spectrogram is a visual representation of the signal strength, or "loudness," of a signal across time at different frequencies contained in a specific waveform. The estimation of spectrograms involves transforming a signal from the time domain to the frequency domain to visualize its frequency content over time using the technique Short-Time Fourier Transform (STFT). The STFT divides the signal into short, overlapping segments, applies the Fourier Transform to each segment, and then combines the results to create a time-varying representation of the signal's frequency content. This process allows for the visualization of how the signal's energy is distributed across different frequencies at each point in time. The horizontal axis in a spectrogram depicts time in the same way that the waveform does, but the vertical axis represents sound frequency, with low frequencies at the bottom and high frequencies at the top. The brightness at that place represents the magnitude of a certain frequency at that time^22^. In addition to this, the Short-Time Fourier Transform (STFT) approach defines three crucial parameters which have been used for this work: `n_fft`, `hop_length`, and `win_length`. The value of the `n_fft` option is 2048, which specifies the size of the analysis window and affects the frequency resolution of the STFT. The `hop_length` option, set to 512, determines the number of samples by which the analysis window moves forward between each frame. This parameter affects the temporal resolution and the amount of overlap between frames. Likewise, the `win_length` parameter is assigned a value of `None`, which means that it will use the default value of `n_fft`. The selected values for these parameters strike a balance between the frequency and time resolution, which affect the properties of the spectral features obtained from the audio stream in later processing stages.

**Figure 3:**
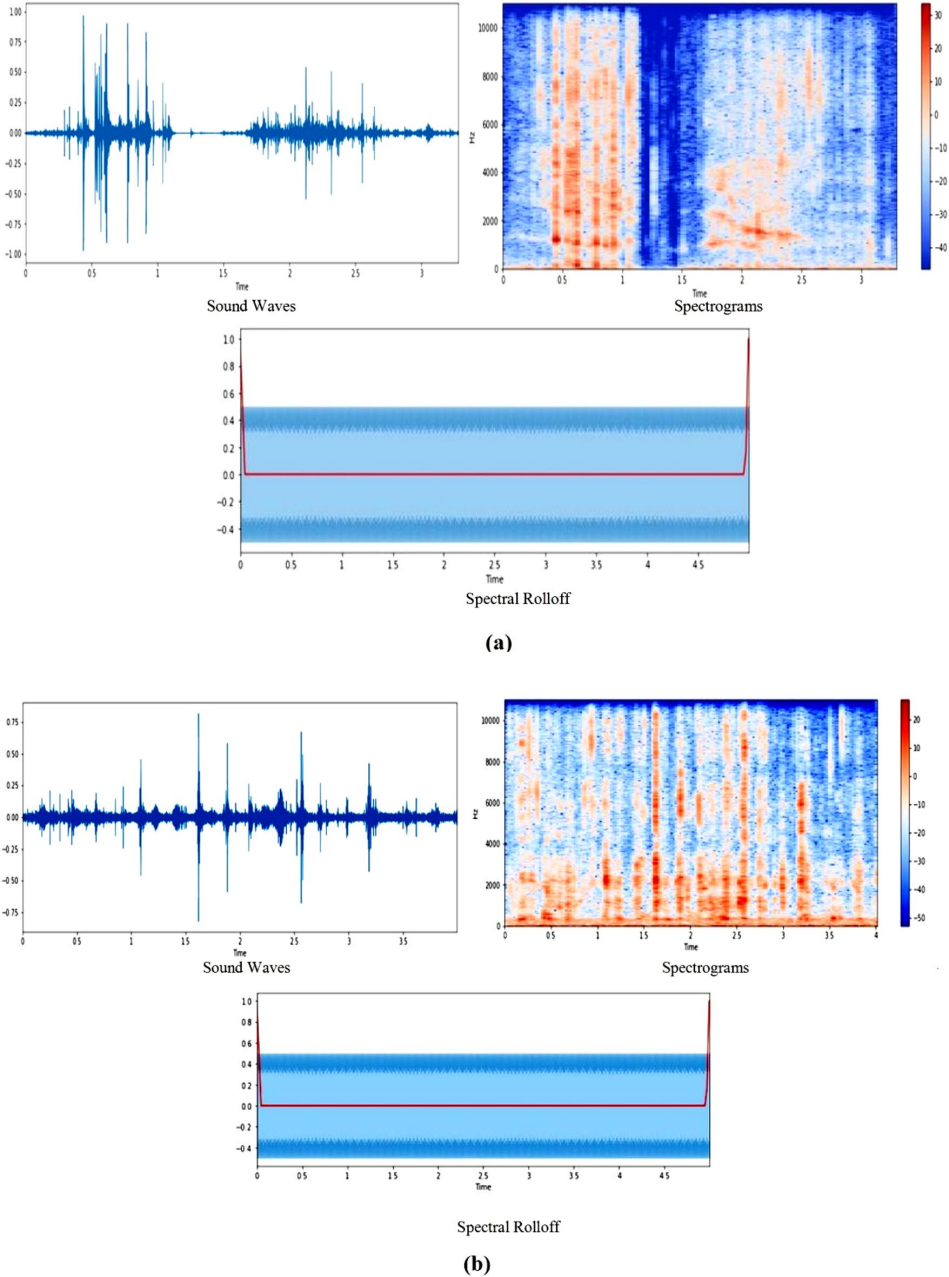

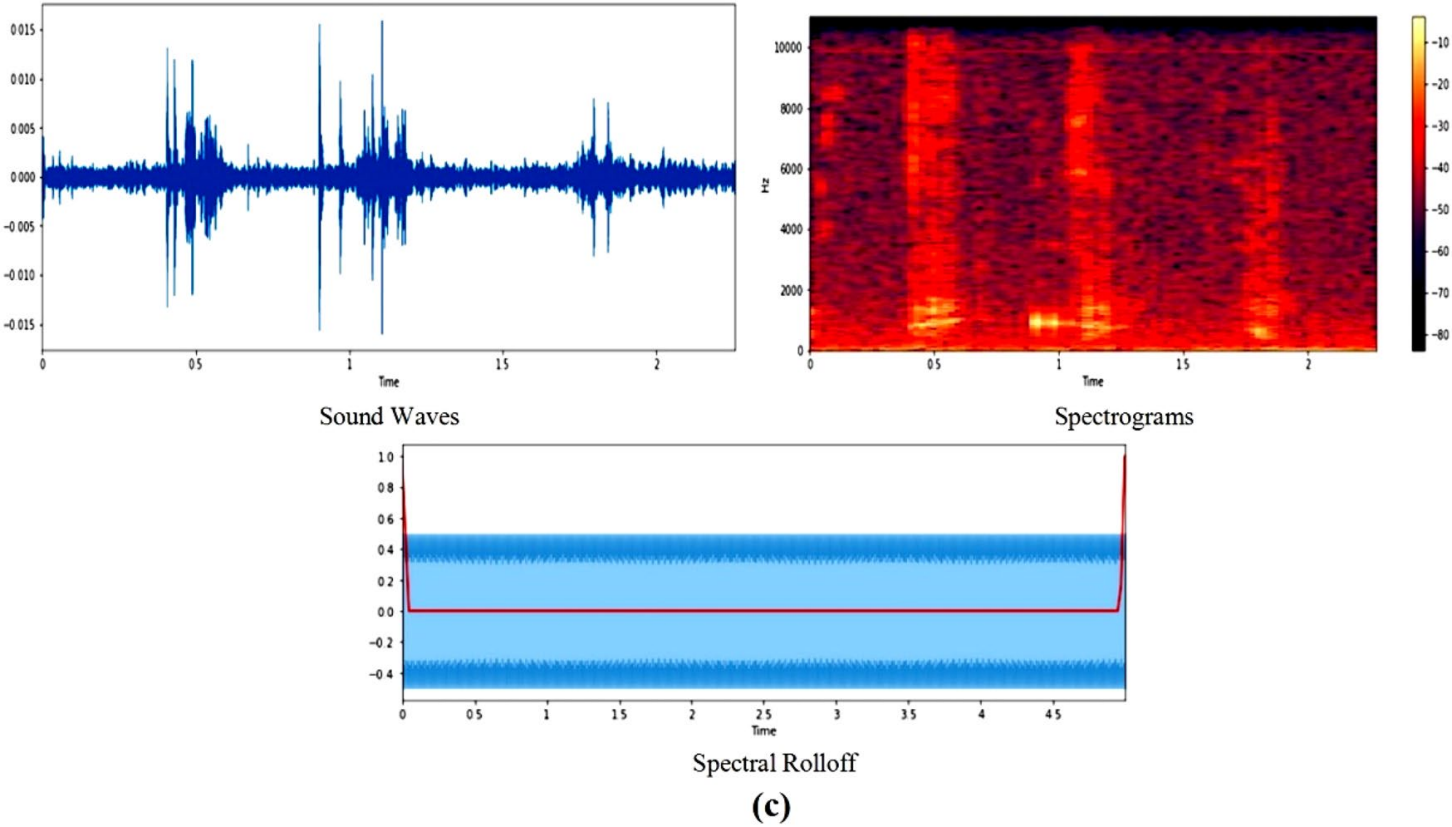
Spectrograms and spectral rolloff generations for food sounds.

Spectral rolloff, on the other hand, is a signal shape measure that represents the frequency at which high frequencies drop to zero. To get it, we calculate the fraction of bins in the power spectrum that have 85% of their power at lower frequencies. To compute the rolloff frequency for each frame of food sounds, the librosa.feature.spectral_rolloff module is employed. Librosa is a Python package for music and audio analysis, providing functions for feature extraction, including spectrogram generation. It impact the proposed approach by providing efficient and user-friendly tools for analyzing and visualizing the audio data, contributing to a more comprehensive understanding of the features extracted from the food sound wave files. Figure 3 shows a sample of food sound wave files together with their spectrograms and spectral rolloff.

#### Spectral bandwidth and MFCCs

The spectral bandwidth, denoted by λ_SB_ and represented on the wavelength axis by two vertical red lines, is akin to the band width of light at half maximum. For a food noise WAV file, its bandwidth is defined as the disparity between the lower and upper frequencies within a continuous frequency range. In the context of signals oscillating around a specific point, Equation (1) encapsulates the concept of spectral bandwidth. It calculates the bandwidth as the sum of the largest deviations of the signal on both sides of the central frequency at a given time frame^23^. This equation serves to quantify the cumulative span of frequencies around the central point, providing a meaningful measure of the signal's spectral characteristics.

1 $${\uplambda }_{{\text{SB}}}= \sum_{i}|{f}_{i}-{f}_{center}|.$$

${f}_{i}$ represents individual frequencies within the continuous range, ${f}_{center}$ is the central frequency around which the signal oscillates, and $\sum_{i}is$ the summation is performed over all relevant frequencies within the considered range.

**Figure 4:**
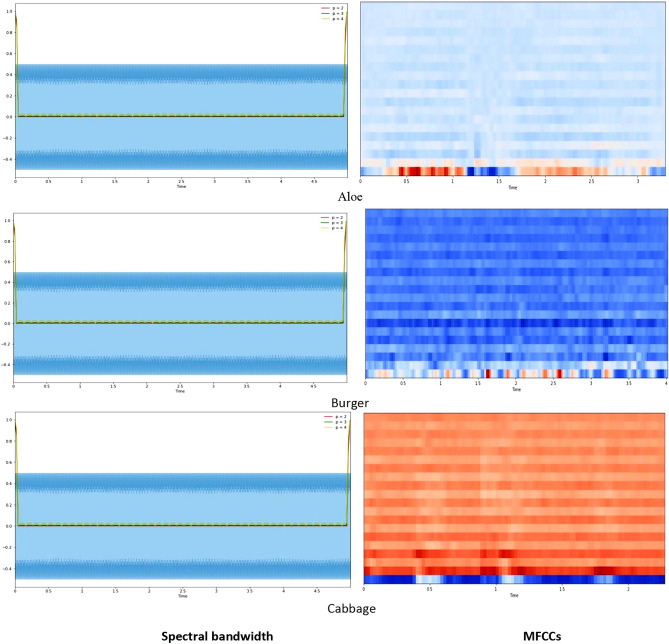
Spectral bandwidth and MFCCs for food sounds.

On the other hand, Mel-frequency cepstral coefficients (MFCCs) play a crucial role in representing timbral and textural aspects of sound, particularly in the context of audio processing in deep learning applications. To extract MFCCs, a Fourier Transform is employed to transition from the time domain to the frequency domain, thus converting the audio signal into a representation suitable for further analysis. The MFCCs, being frequency domain features, provide a nuanced understanding of the underlying audio characteristics. The process involves several steps, starting with the division of the audio signal into frames. Let $x(t)$ represents the audio signal at time $t$, and $X(\omega )$ denotes its Fourier transform. The power spectrum $S(\omega )$ is computed as ${\left|X(\omega )\right|}^{2}$. Following this, a filter bank is applied and the logarithm of the filter bank energies is calculated. The Discrete Cosine Transform (DCT) is then employed to obtain the final MFCCs. Mathematically, the $i-th$ MFCC coefficient, ${c}_{i}$, is expressed as shown in Equation (2):

2 $${c}_{i}= \sum_{j=1}^{N}{\text{log}}\left({S}_{j}\right).{\text{cos}}\left(\frac{\pi i\left(j-0.5\right)}{N}\right),$$

where ${S}_{j}$ represents the energy in the $j-th$ filter of the filter bank, and N is the total number of filters. This process is conducted for each frame, resulting in a time sequence of MFCC vectors, capturing the evolution of these coefficients over time. The number of coefficients, typically ranging from 13 to 40, can be adjusted based on the desired level of feature granularity. Overall, the extraction of MFCCs provides a comprehensive representation of the frequency content of audio signals, crucial for effective utilization in deep learning models aimed at understanding and processing auditory information. Further, the obtained data is then split in to training and validation in the form of 75% and 25% respectively. Figure 4 represent the spectral bandwidth and MFCC for food sounds:

### Applied models

Once the required features have been extracted, various deep learning classifiers have been trained for their efficacy in capturing temporal patterns in sequential audio data. In fact, these applied deep learning models play a crucial role in recognizing and classifying various food-related sounds. Throughout the training process, a categorical cross-entropy loss function, 0.0001 learning rate, batch size of 32, and the number of epochs (20) for deep transfer learning models and 200 epochs in case of customized CNN model during training has been employed to optimize the models for accurate classification. This approach aimed to leverage the strengths of recurrent neural networks in handling sequential data which contributes to the effective detection and classification of food sounds in the dataset.

LSTM (Long Short Term Memory) is a type of recurrent neural network developed to overcome the vanishing gradient problem that frequently happens during deep neural network training. The core concept of LSTM is to employ memory cells and gates to selectively recall or forget information from earlier time steps. Each LSTM cell has three gates which are used for controlling the flow of information: the input gate ($${I}_{T})$$, the forget gate ($${F}_{T})$$, and the output gate ($${O}_{T})$$.

The input and the previous cell state ${(C}_{T-1})$ are sent via the input gate at each time step, which determines how much of the new input to remember. The prior hidden state ${h}_{T}$ and the input are both sent via the forget gate, which determines how much of the previous hidden state should be forgotten. The changed hidden state is then used to calculate the output, which is then transmitted through the output gate. In addition to the gates, the LSTM contains a memory cell that functions as a "conveyor belt," allowing information to be transported across time steps without being altered. The input and forget gates can also modify the memory cell, allowing the network to selectively store or delete information as required^24–26^. It can be mathematically computed by using Equations ((3), (4), and (5)).

3 $${I}_{T}=\sigma \left({W}_{I}\left[{h}_{T-1},{X}_{t}\right]+{b}_{I}\right)$$

4 $${F}_{T}=\sigma \left({W}_{F}\left[{h}_{T-1},{X}_{t}\right]+{b}_{F}\right)$$

5 $${O}_{T}=\sigma \left({W}_{O}\left[{h}_{T-1},{X}_{t}\right]+{b}_{O}\right)$$

Here ${W}_{X}$—the weight of all gates,—$\sigma$sigmoid function, ${h}_{T-1}$—output of the previous LSTM block at timestamp (T-1), X—neurons, ${b}_{X}$—biases for respective gates, and ${X}_{T}$—input at current timestamp.

In this research work, the model begins with an LSTM layer with an output shape of (None, None, 128), followed by another LSTM layer producing an output shape of (None, 64), as mentioned in Table 2. The subsequent Dense layer yields an output shape of (None, 64), which is then passed through a Dropout layer resulting in an output shape of (None, 64). Finally, a dense layer produces an output shape of (None, 20). The term "None" in the output shape column signifies that the corresponding dimension is not fixed or constrained to a specific size.

**Table 2:** Parameters of LSTM.

Number of layers	Name of layers	Output shape
1	LSTM	(None, None, 128)
2	LSTM	(None, 64)
3	Dense	(None, 64)
4	Dropout	(None, 64)
5	Dense	(None, 20)

GRU (Gated Recurrent Unit) is also a type of recurrent neural network and is similar to long short term memory but has fewer parameters and is computationally less expensive than LSTM. The main idea behind GRU is to use gating mechanisms for selectively remembering or forgetting the information from previous time steps. GRU has two types of gates, a reset gate (R) and an update gate (Z), which control the flow of information. At each time step, the input (*X)* and the previous hidden state are passed through the reset gate, which decides how much of the previous hidden state to forget. Then, the input and the modified previous hidden state are passed through the update gate, which decides how much of the new input to remember. The output is then calculated based on the modified hidden state^27,28^. Mathematically, it can be computed by using Equations ((6), (7), (8), and (9)).

6 $${Z}_{t}= \sigma \left({W}_{z} .\left[{H}_{t-1}, {X}_{t}\right]\right)$$

7 $${R}_{t}= \sigma \left({W}_{z} .\left[{H}_{t-1}, {X}_{t}\right]\right)$$

8 $${\widetilde{H}}_{t}={\text{tanh}}(W . [{R}_{t}* {H}_{t-1}, {x}_{t}])$$

9 $${H}_{t}=\left(1-{Z}_{t}\right)* {H}_{t-1}+ {Z}_{t}*{\widetilde{h}}_{t},$$

where H and $\widetilde{H}$ and represent the output and candidate hidden state respectively, $\sigma$ is an activation function, ${Z}_{t}$ is update gate at time step *t*, ${\text{tanh}}$ is the hyperbolic tangent activation function, ${W}_{z}$ is weight matrix associated with update gate, and $W$ is the weight of the candidate hidden state.

In this research, the model begins with a GRU layer with an output shape of (None, None, 128), followed by another GRU layer producing an output shape of (None, 64), as shown in Table 3. The subsequent Dense layer yields an output shape of (None, 64), which is then passed through a Dropout layer resulting in an output shape of (None, 64). Finally, a Dense layer produces an output shape of (None, 20). [In the output shapes column, 'None' represents a flexible or variable dimension that can vary based on the input data].

**Table 3:** Parameters of GRU model.

Number of layers	Name of layers	Output shape
1	GRU	(None, None, 128)
2	GRU	(None, 64)
3	Dense	(None, 64)
4	Dropout	(None, 64)
5	Dense	(None, 20)

A bidirectional GRU (gated recurrent unit) is a type of recurrent neural network (RNN) that processes sequential data in both backward and forward directions. In a bidirectional GRU, there are two GRU layers in which one sequence is processed in the forward direction and the other is processed in the backward direction. The output of each layer at each time step is concatenated to form a final output which is enabling the model to incorporate both future and past context while predicting any task or event^22^. The formulae to compute this network is shown in Equations ((10), (11), and (12)).

10 $$\overrightarrow{{H}_{t}}={GRU}_{fwd} ({X}_{t}, \overrightarrow{{H}_{t-1}})$$

11 $$\overleftarrow{{H}_{t}}= {GRU}_{bwd} ({X}_{t} , \overleftarrow{{H}_{t+1}} )$$

12 $${H}_{t}= \overrightarrow{{H}_{t}} \oplus \overleftarrow{{H}_{t}}$$

where $\overleftarrow{{H}_{t}}$ is the backward state GRU, $\overrightarrow{{H}_{t}}$ is the forward state GRU, ⊕ indicates the concatenation operation of two vectors, ${X}_{t}$ is input at time t^29,30^.

In this study, we combined RNN with bidirectional GRU to accomplish food detection and classification and its layered architecture is shown in Table 4. The first layer is a dense layer, which is a fully linked layer in which every neuron in the previous layer is connected to every neuron in the current layer. Despite the layer having 128 neurons, it produces an output form of (None, None, 128), which demonstrates that the batch size and sequence length can vary. A SimpleRNN layer, a kind of recurrent layer that enables the network to recall previous inputs, is the second layer. It generates the shape (None, 128), indicating that the batch size is flexible and that there are 128 neurons in the layer. A bidirectional GRU layer, or bidirectional recurrent layer using the GRU architecture, is the third layer. Bidirectional layers analyze the input sequence in both a forward and a reverse orientation, collecting data from the past and the future. With a batch size that is user-configurable and a layer of 64 neurons, this layer generates a variant of (None, 64). The fourth layer is another dense layer that is fully connected and has an output shape of (None, 64). It has 64 neurons and is linked to the previous layer. The fifth layer is a dropout layer, which prevents overfitting by randomly removing a percentage of input units during training. It keeps the previous layer's shape, resulting in an output shape of (None, 64). Finally, the sixth layer is another dense layer with a (None, 20) output form. It is a fully connected layer with 20 neurons that produces the model's final output.

**Table 4:** Parameters of RNN + BidirectionalGRU.

Number of layers	Name of layers	Output shape
1	Dense	(None, None, 128)
2	SimpleRNN	(None, 128)
3	Bidirectional GRU	(None, 64)
4	Dense	(None, 64)
5	Dropout	(None, 64)
6	Dense	(None, 20)

Bidirectional Long Short-Term Memory (BiLSTM) is used for sequence data processing. In a conventional LSTM model, the input sequence is processed sequentially in a unidirectional fashion, often from left to right. Each time step's output is then used as input for the next time step. The BiLSTM model, on the other hand, processes the input sequence in a bidirectional way. This is accomplished by separating the sequence into two independent sequences, one processed forward (from left to right) and the other backward (from right to left)^31^. The BiLSTM can collect information from both past and future events. This is because the hidden states at each time step are influenced by both the previous and next time steps. The fusion of forward and backward hidden states is commonly achieved through concatenation, which yields a combined hidden state that integrates information from both directions. The fused hidden state is utilized for making predictions or transmitting to subsequent layers within the neural network architecture for additional processing^32,33^. Let $X=({x}_{1}, {x}_{2}, \dots \dots , {x}_{T})$ be the input sequence of the length $T$, where ${x}_{t}$ is the input at time step $t$. The forward hidden states ${h}_{t}^{forward}$ are computed as shown in Eq. (13):

13 $${h}_{t}^{forward}=LSTM\left({x}_{t}, {h}_{t-1}^{forward}\right).$$

Here, $LSTM$ is the operation performed by the Long Short-Term Memory cell, which involves computations like input and output gate activations, cell state updates, and hidden state computations. The final output at each time step $t$ is a concatenation of the forward and backward hidden states, as shown in Eq. (14):

14 $${h}_{t}=\left[{h}_{t}^{forward};{h}_{t}^{backward}\right]$$

The semicolon $(;)$ denotes concatenation. The output sequence $H =({h}_{1}, {h}_{2}, \dots \dots , {h}_{T})$ can be used for further tasks such as classification or sequence-to-sequence prediction.

In this study, we combined RNN with bidirectional LSTM to accomplish food sound dataset detection and classification. Table 5 depicts a neural network model with several layers and their associated output shapes. Each layer has a distinct role to play in processing and modifying the input data. The dense layer is a completely connected layer that transforms the input data linearly by linking every neuron from its preceding layer to succeeding layer. The output shape of this layer is (None, None, 128) where None indicates that the dimension can change depending on the input information. The number "128" designates the layer's total number of neurons. A recurrent layer built with SimpleRNN units is called the SimpleRNN Layer. Its purpose is to manage sequential data and maintain data from prior inputs. This layer gives a fixed-size output for each input sequence because its output shape is (None, 128). The long-short-term memory (LSTM) layer type known as the bidirectional LSTM layer processes the input sequence both forward and backward. The output shape for this layer is (None, 64), which denotes a fixed-size output. Like the preceding layer, this one is also dense and totally connected. It applies a linear transformation to the output of the layer that came before it. The output shape is identical to the preceding layer (None, 64). Dropout is a regularization technique that helps to lessen the network's reliance on specific features and encourages it to learn more robust representations. The output shape is unchanged (None, 64). The model's last dense layer applies additional linear transformations to the input and generates the final output. The output shape is (None, 20), which indicates that it has 20 neurons, which may correspond to different groups or categories depending on the task.

**Table 5:** Parameters of RNN + BidirectionalLSTM.

Number of layers	Name of layers	Output shape
1	Dense	(None, None, 128)
2	SimpleRNN	(None, 128)
3	Bidirectional LSTM	(None, 64)
4	Dense	(None, 64)
5	Dropout	(None, 64)
6	Dense	(None, 20)

The InceptionResNetV2 architecture combines the Inception and ResNet modules to form a deep convolutional neural network (CNN). By using residual connections within the Inception module, InceptionResNetV2 merges these two components^34^. It also has various other features to increase network performance and stability, such as batch normalization, dropout, and pre-activation. InceptionResNetV2's architecture comprises of several levels, with a total of 164 layers, including multiple Inception and ResNet modules^35–38^.

The neural network model depicted in Table 6 has four layers. The first layer is an Inception ResNetV2 layer that extracts features from input data using a combination of Inception and ResNet modules. It yields the shape (None, 5, 5, 1536), suggesting a feature map with a spatial size of 5 × 5 and 1536 channels. The second layer is a global average pooling layer that shrinks the spatial dimensions and outputs a single value for each channel, yielding an output shape of (None, 1536).The third layer is a Dropout layer and its output shape remains the same, (None, 1536). Finally, there is a Dense layer, a fully connected layer that produces the final output with a shape of (None, 20), which implies 20 neurons representing different classes or categories depending on the specific task. [In the output shapes column, 'None' represents a flexible or variable dimension that can vary based on the input data].

**Table 6:** Parameters of InceptionResNetV2.

Number of layers	Name of layers	Output shape
1	Inception ResNetV2	(None, 5, 5, 1536)
2	Global Average Pooling	(None, 1536)
3	Dropout	(None, 1536)
4	Dense	(None, 20)

Besides applying the advanced deep learning models, we have also customized the CNN model to detect and classify the eating sound of various food items. Table 7 represents a neural network model with different layers and their corresponding output shapes. It starts with the MFCC input layer where MFCC stands for Mel Frequency Cepstral Coefficients, which are commonly used features for audio processing. This layer represents the input layer of the network and has an output shape of (None, 64), where "None" indicates that the batch size can vary, and 64 represents the number of MFCC coefficients. Subsequently, there is a *Dense Layer* which is a fully connected layer applies a linear transformation to the input data. It relies on the output from the preceding layer and has 512 neurons, as indicated by its output shape of (None, 512). *Batch Normalization* technique is used for normalizing the activations of a neural network layer. It helps to stabilize and improve the training process. The output shape remains the same as the previous layer i.e. (None, 512). Next is the *Activation Relu* where the activation function is applied element-wise to the output of the previous layer. ReLU (Rectified Linear Unit) is a commonly used activation function that introduces non-linearity to the model. Further, there is a *Dropout Layer* which is a regularization technique which sets a fraction of input units to 0 randomly during training. It prevents overfitting by reducing the reliance on specific features. The output shape remains the same as the previous layer, (None, 512).

**Table 7:** Parameters of CustomizedCNN.

Layers	Output shape	Parameters
MFCC input layer	(None, 64)	0
Dense layer	(None, 512)	33,280
Batch normalization	(None, 512)	2048
Activation Relu	(None, 512)	0
Dropout layer	(None, 512)	0
Dense layer	(None, 256)	131,328
Batch normalization	(None, 256)	1024
Activation Relu	(None, 256)	0
Dropout layer	(None, 256)	0
Dense layer	(None, 20)	5140

Another fully connected layer *(Dense Layer)* with an output shape of (None, 512) and is succeeded by another *batch normalization layer* with the same output shape as the previous layer (None, 512) as well as ReLU activation function. Likewise, another *dropout layer* with the same output shape as the previous layer is applied and is finally concluded by final *fully connected layer* with an output shape of (None, 20) which indicates 20 neurons representing different classes or categories depending on the specific task.

The values provided in the last column represent the number of parameters (weights and biases) in each layer. For example, "0" indicates that the MFCC Input Layer does not have any learnable parameters, while "33280" represents the number of parameters in the Dense Layer. It's important to note that the number of parameters in a layer depends on the size of its input and output dimensions.

### Evaluative parameters

Performance evaluation metrics such as accuracy, loss, precision, recall, and F1 score play a pivotal role in assessing the effectiveness of machine learning models. These metrics are valuable tools for model comparison and fine-tuning of hyperparameters to enhance overall performance^18,39–41^. The following Equations ((15), (16), (17), (18), (19)) are commonly employed to quantify these metrics and analyze the performance of diverse machine learning models.

15 $$Accuracy= \frac{True\, Positive+True\, Negative}{True\, Positive+True\, Negative+False \,Positive+False \,Negative}$$

16 $$Loss=\frac{{(Actual \,Value-Predicted \,Value)}^{2}}{Number \,of\, observations}$$

17 $$Precision \left({\text{Pr}}\right)=\frac{True\, Positive}{True \,Positive+False\, Positive}$$

18 $$Recall \left({\text{Re}}\right)=\frac{True\,positive}{True\,positive+False\,Negative}$$

19 $$F1 \,score\, (F1)= 2\frac{Precision*Recall}{Recall+Precision}$$

## Results

In this section the various learning models such as GRU, LSTM, Bidirectional LSTM + GRU, Simple RNN + Bidirectional GRU, Simple RNN + Bidirectional LSTM, InceptionResNetV2 including the customized CNN that have been trained with the dataset are evaluated and displayed based on the parameters as mentioned in Sect. "Evaluative parameters". In the initial evaluation phase, models are assessed for accuracy and data loss, with subsequent scrutiny of precision, recall, and F1 score across the entire dataset and its individual classes.

From Table 8, during training phase, the GRU model exhibited the highest accuracy at 96.45%, closely followed by the CNN model at 96.62%. Both models achieved low loss values (0.11 for GRU and 0.09 for CNN), indicating strong predictive capabilities and efficient convergence during training. The Bidirectional LSTM + GRU model also demonstrated competitive performance with an accuracy of 95.77% and a relatively low loss of 0.13. Interestingly, the InceptionResNetV2, designed for image classification, showcased notable adaptability with a commendable accuracy of 95.61%. However, the Simple RNN-based architectures, both standalone and in combination with Bidirectional LSTM or GRU, exhibited slightly lower accuracies, suggesting that the more advanced recurrent and convolutional architectures better capture the intricate temporal patterns present in food-related sounds, leading to improved classification performance.

**Table 8:** Accuracy and loss values of models.

Model	Training		Validation	
	Accuracy (%)	Loss	Accuracy (%)	Loss
LSTM	94.46	0.17	95.57	0.15
GRU	96.45	0.11	99.28	0.02
Bidirectional LSTM + GRU	95.77	0.13	98.27	0.06
Simple RNN + Bidirectional GRU	92.07	0.25	97.48	0.08
Simple RNN + Bidirectional LSTM	94.03	0.17	97.83	0.07
InceptionResNetV2	95.61	0.29	94.56	0.64
CNN	96.62	0.09	95.96	0.15

On the contrary, in validation phase, the GRU model stands out with an exceptionally high accuracy of 99.28% and a remarkably low loss of 0.02, indicating robust generalization capabilities. The Bidirectional LSTM + GRU and Simple RNN + Bidirectional LSTM models also exhibit strong performance with accuracies of 98.27% and 97.83%, respectively, and relatively low losses. The LSTM model maintains a solid performance with an accuracy of 95.57% and a moderate loss of 0.15. The Simple RNN + Bidirectional GRU model achieves an accuracy of 97.48%, while both the InceptionResNetV2 and CNN models, originally designed for image classification, demonstrate reasonable adaptability with accuracies of 94.56% and 95.96%, respectively. The consistency in performance across training and validation phases underscores the models' ability to effectively generalize to unseen data, with GRU showcasing particularly impressive results in this regard.

Similarly, as mentioned earlier, the models have been also examined for another set of parameters i.e. precision, F1 score, and recall as shown in Table 9. The presented models exhibit varying levels of performance across precision, recall, and F1 score metrics. The Bidirectional LSTM + GRU model demonstrates strong overall performance, achieving high precision of 97.7%, 97% recall, and 97.3% F1 Score. The GRU model follows closely, displaying notable 96.2% precision and 97.3% recall, resulting in a commendable F1 score of 97.2%. The Simple RNN + Bidirectional GRU model also performs well with balanced 96.8% precision and 97.1% recall, yielding a competitive F1 score of 96.75%. Conversely, the Simple RNN + Bidirectional LSTM model, while achieving respectable precision and recall, falls slightly short in the F1 score at 96.95%. The InceptionResNetV2 model demonstrates a comparatively lower F1 score of 93.2% despite reasonably high precision of 94.15% and recall of 96.65%. The CNN model, while achieving a high precision of 97.15%, experiences a minor dip in recall (96.75%) and F1 score (96.55%). Overall, the Bidirectional LSTM + GRU and GRU models stand out as top performers in this evaluation which implies higher performance in this task. However, other models also display reasonably decent performance.

**Table 9:** Performance analysis of models.

Model	Precision	Recall	F1 score
LSTM	95.15	95.35	95.2
GRU	96.2	97.3	97.2
Bidirectional LSTM + GRU	97.7	97	97.3
Simple RNN + Bidirectional GRU	96.8	97.1	96.75
Simple RNN + Bidirectional LSTM	96.55	97.45	96.95
InceptionResNetV2	94.15	96.65	93.2
CNN	97.15	96.75	96.55

**Figure 5:**
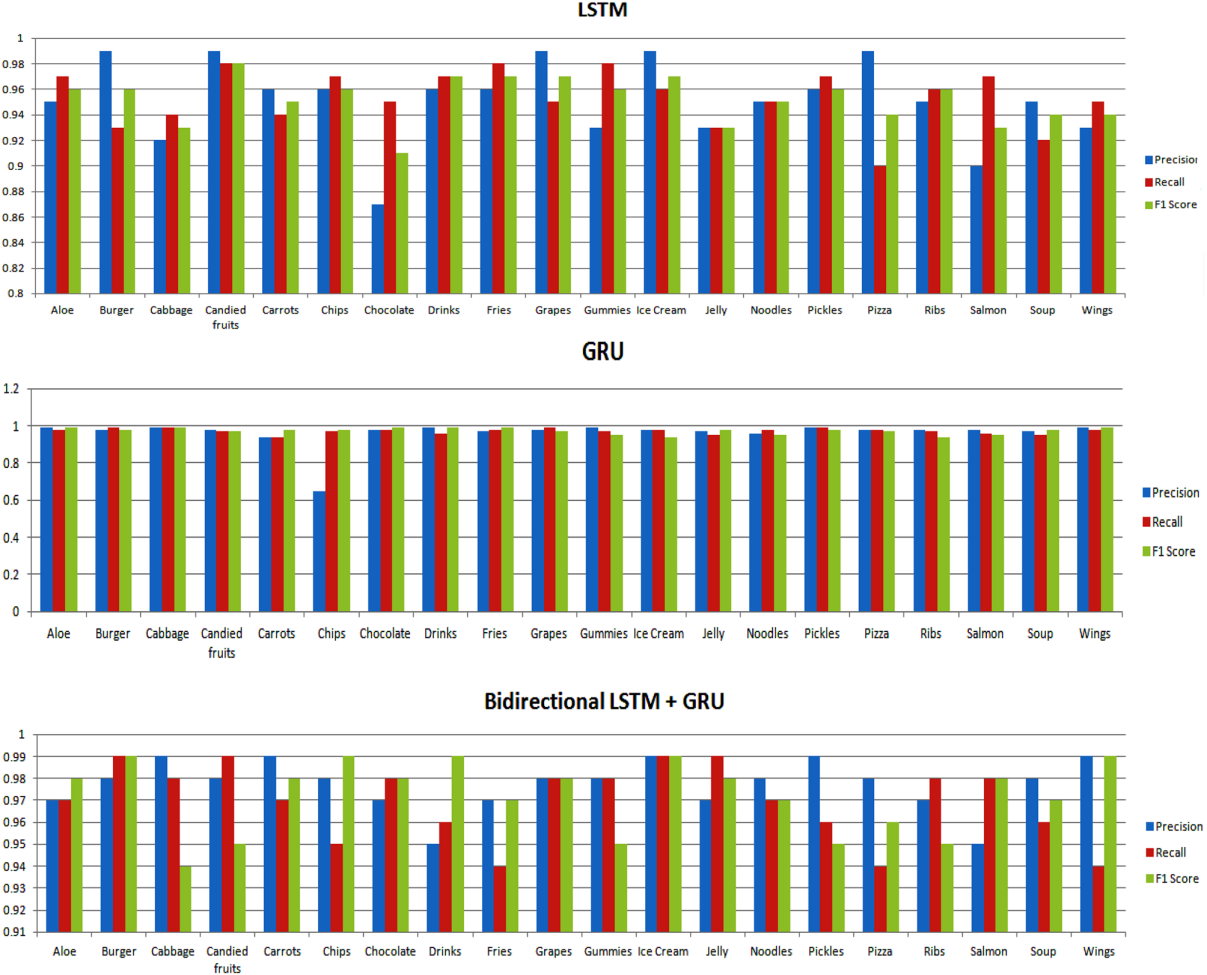

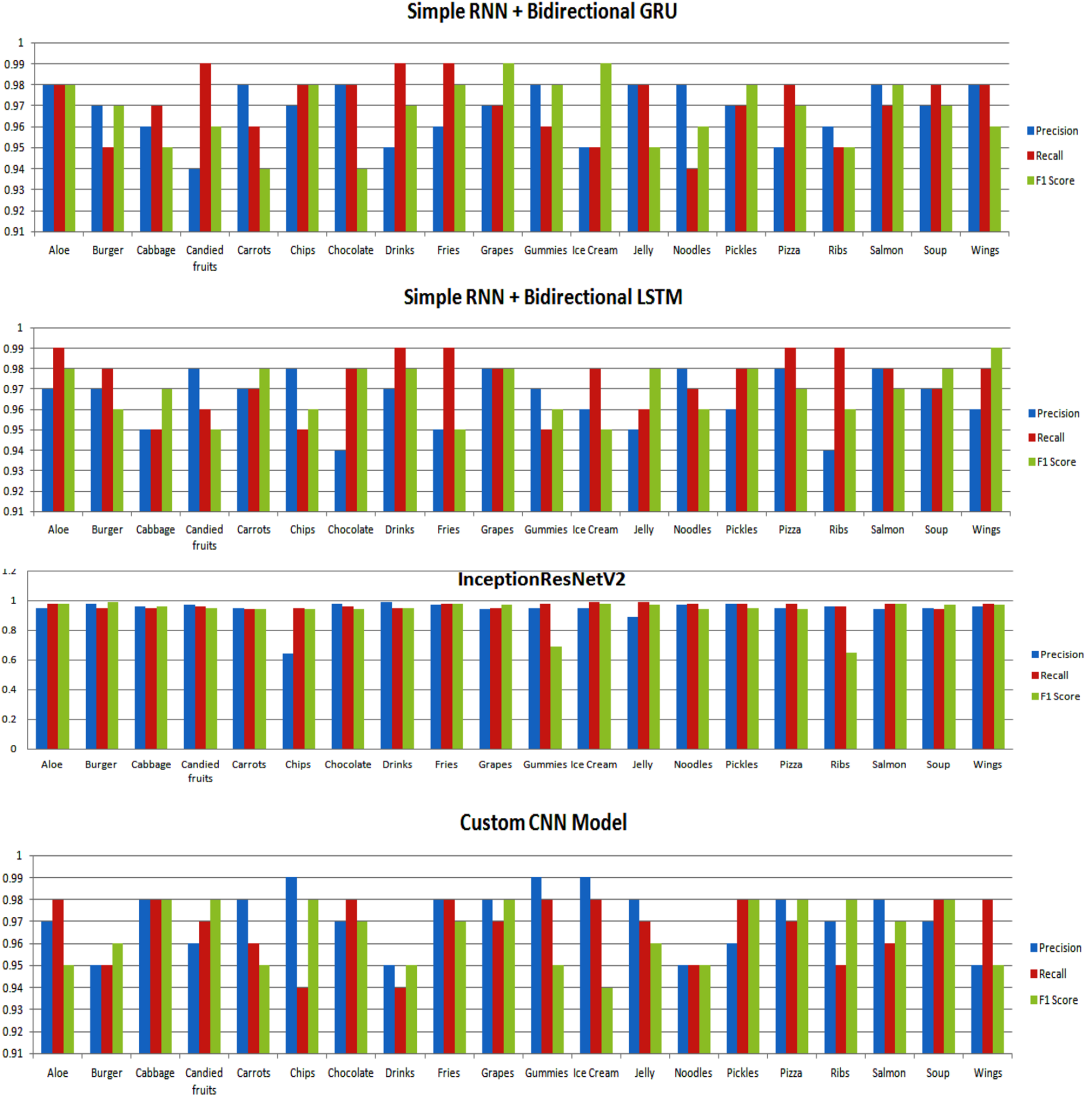
Performance evaluation of deep learning models.

Furthermore, the evaluation extends to the classification models when trained on a dataset comprising twenty distinct classes. The performance is thoroughly scrutinized using various metrics such as precision, recall, and F1 score, as depicted in Fig. 5. Additionally, for clarity and detailed reference, a tabular representation of these results is presented in Table 10. This comprehensive analysis allows for a nuanced understanding of each model's efficacy in handling a diverse set of twenty classes, providing valuable insights into their performance across multiple metrics.

**Table 10:** Analysing the performance of models for various classes.

Classes	LSTM			GRU			BidirectionalLSTM + GRU			RNN + BidirectionalGRU			RNN + BidirectionalLSTM			InceptionResNetV2			Customized CNN		
	Pr	Re	F1	Pr	Re	F1	Pr	Re	F1	Pr	Re	F1	Pr	Re	F1	Pr	Re	F1	Pr	Re	F1
Aloe	0.95	0.97	0.96	0.99	0.98	0.99	0.97	0.97	0.98	0.98	0.98	0.98	0.97	0.99	0.98	0.95	0.98	0.98	0.97	0.98	0.95
Burger	0.99	0.93	0.96	0.98	0.99	0.98	0.98	0.99	0.99	0.97	0.95	0.97	0.97	0.98	0.96	0.98	0.95	0.99	0.95	0.95	0.96
Cabbage	0.92	0.94	0.93	0.99	0.99	0.99	0.99	0.98	0.94	0.96	0.97	0.95	0.95	0.95	0.97	0.96	0.95	0.96	0.98	0.98	0.98
Candied fruits	0.99	0.98	0.98	0.98	0.97	0.97	0.98	0.99	0.95	0.94	0.99	0.96	0.98	0.96	0.95	0.97	0.96	0.95	0.96	0.97	0.98
Carrots	0.96	0.94	0.95	0.94	0.94	0.98	0.99	0.97	0.98	0.98	0.96	0.94	0.97	0.97	0.98	0.95	0.94	0.94	0.98	0.96	0.95
Chips	0.96	0.97	0.96	0.65	0.97	0.98	0.98	0.95	0.99	0.97	0.98	0.98	0.98	0.95	0.96	0.64	0.95	0.94	0.99	0.94	0.98
Chocolate	0.87	0.95	0.91	0.98	0.98	0.99	0.97	0.98	0.98	0.98	0.98	0.94	0.94	0.98	0.98	0.98	0.96	0.94	0.97	0.98	0.97
Drinks	0.96	0.97	0.97	0.99	0.96	0.99	0.95	0.96	0.99	0.95	0.99	0.97	0.97	0.99	0.98	0.99	0.95	0.95	0.95	0.94	0.95
Fries	0.96	0.98	0.97	0.97	0.98	0.99	0.97	0.94	0.97	0.96	0.99	0.98	0.95	0.99	0.95	0.97	0.98	0.98	0.98	0.98	0.97
Grapes	0.99	0.95	0.97	0.98	0.99	0.97	0.98	0.98	0.98	0.97	0.97	0.99	0.98	0.98	0.98	0.94	0.95	0.97	0.98	0.97	0.98
Gummies	0.93	0.98	0.96	0.99	0.97	0.95	0.98	0.98	0.95	0.98	0.96	0.98	0.97	0.95	0.96	0.95	0.98	0.69	0.99	0.98	0.95
Ice Cream	0.99	0.96	0.97	0.98	0.98	0.94	0.99	0.99	0.99	0.95	0.95	0.99	0.96	0.98	0.95	0.95	0.99	0.98	0.99	0.98	0.94
Jelly	0.93	0.93	0.93	0.97	0.95	0.98	0.97	0.99	0.98	0.98	0.98	0.95	0.95	0.96	0.98	0.89	0.99	0.97	0.98	0.97	0.96
Noodles	0.95	0.95	0.95	0.96	0.98	0.95	0.98	0.97	0.97	0.98	0.94	0.96	0.98	0.97	0.96	0.97	0.98	0.94	0.95	0.95	0.95
Pickles	0.96	0.97	0.96	0.99	0.99	0.98	0.99	0.96	0.95	0.97	0.97	0.98	0.96	0.98	0.98	0.98	0.98	0.95	0.96	0.98	0.98
Pizza	0.99	0.90	0.94	0.98	0.98	0.97	0.98	0.94	0.96	0.95	0.98	0.97	0.98	0.99	0.97	0.95	0.98	0.94	0.98	0.97	0.98
Ribs	0.95	0.96	0.96	0.98	0.97	0.94	0.97	0.98	0.95	0.96	0.95	0.95	0.94	0.99	0.96	0.96	0.96	0.65	0.97	0.95	0.98
Salmon	0.90	0.97	0.93	0.98	0.96	0.95	0.95	0.98	0.98	0.98	0.97	0.98	0.98	0.98	0.97	0.94	0.98	0.98	0.98	0.96	0.97
Soup	0.95	0.92	0.94	0.97	0.95	0.98	0.98	0.96	0.97	0.97	0.98	0.97	0.97	0.97	0.98	0.95	0.94	0.97	0.97	0.98	0.98
Wings	0.93	0.95	0.94	0.99	0.98	0.99	0.99	0.94	0.99	0.98	0.98	0.96	0.96	0.98	0.99	0.96	0.98	0.97	0.95	0.98	0.95

Based on the results, the *LSTM* model has attained good precision and recall ratings for the majority of the food categories. Precision ranges from 0.87 to 0.99, recall ranges from 0.90 to 0.98, and F1 ranges from 0.91 to 0.98. Based on these data, it can be stated that the model provides adequate precision and recall rates for the majority of food categories. There are differences in performance measures across food categories, with certain categories scoring lower in specific metrics. Pizza, for example, has a lesser recall (0.90), whereas chocolate has a lower precision (0.87). These variances indicate potential areas for model improvement or fine-tuning to improve overall performance across all food groups.

With precision and recall values greater than 0.95 and an F1 score larger than 0.97, the *GRU* model performs well in culinary categories such as aloe, cabbage, candied fruits, carrots, chocolate, drinks, fries, grapes, pickles, and wings. This indicates that the model accurately predicts these dietary categories; however, for chips, gummies, ice cream, jelly, noodles, pizza, ribs, salmon, and soup, the model's performance is relatively poor, with precision, recall, and F1 Score values that falls in between 0.65 to 0.98. This implies that the model may contain false positives or false negatives in certain categories and that its efficacy should be improved.

The *bidirectional LSTM* + *GRU* model has been shown to have good precision, recall, and F1 score values that range from 0.94 to 0.99 for each food class. This suggests that the model is capable of properly categorising sound related to these food categories. Other aspects, including the amount and quality of the dataset, model hyper-parameters, and task-specific needs, must be considered when interpreting these results. As evidenced by the model's high performance metrics, the bidirectional LSTM and GRU model is good at classifying food categories based on eating sound.

Likewise, *RNN* + *BidirectionalGRU* demonstrates consistently high precision values for most classes by ranging from 0.94 to 0.98 and indicate a low false positive rate in classifying food items. Additionally, the recall values are generally robust by computing the scores from 0.94 to 0.99 and showcase the model's ability to capture a substantial portion of true positive instances. The F1 scores fall within a narrow range of 0.94 to 0.99 and indicate an overall well-balanced performance across different food categories. It is worth noting that the model excels in discriminating between classes, particularly evident in its ability to distinguish between similar food items like Aloe and Burger.

In evaluating the performance of the *RNN* + *BidirectionalLSTM* model across various categories of the food, the precision values range from 0.94 to 0.98 which indicates a low false-positive rate, while recall values range from 0.95 to 0.99 and highlights the ability of the model's to capture a high percentage of true positives. The F1 score range from 0.95 to 0.99 and demonstrates the overall effectiveness of the model in achieving a balance between precision and recall. The model excels in differentiating between distinct food categories, with notable performance on classes such as Aloe, Drinks, Burger, and Wings. However, slight variations in performance are observed across classes that suggest potential areas for further refinement. Overall, the RNN + BidirectionalLSTM model exhibits a robust as well as competitive performance in the context of food category classification.

Similarly, for classes like Aloe, Cabbage, Burger, Candied fruits, and others, the model exhibits strong precision, recall, and F1 scores by ranging from 0.94 to 0.99. This implies that *InceptionResNetV2* model effectively identifies and classifies instances of these food classes with high accuracy and minimal misclassification. However, for the 'Gummies' and 'Ribs' categories, the precision and recall values are comparatively lower, particularly for 'Gummies,' where the F1 score is also reduced. This indicates that the model may struggle with accurate predictions for these specific food categories.

In the end, on evaluating the performance of the *customized Convolutional Neural Network* (CNN) model to classify food item on the basis of sounds, we observe that the model showcases excellent precision that ranges from 0.95 to 0.99 and indicates a low false-positive rate. The recall values which spans from 0.94 to 0.98, reflects the ability of the model to effectively identify instances of each class and minimize the false negatives. The F1 scores, which balance the precision and recall, range from 0.94 to 0.98 to emphasize the overall robustness of the model. Notably, certain classes such as Chips and Gummies exhibit exceptional performance across all metrics. These results suggest that the customized CNN model effectively discriminates between different food items, showcasing its potential for accurate and reliable classification in a diverse range of scenarios.

## Discussion

The presented research on food identification using deep learning based on eating sounds sparks an interesting debate on the potential uses, problems, and future directions of this novel approach. One of the research's primary features is its capacity to handle different practical concerns with food identification. The suggested technology, which analyses eating sounds, provides a non-intrusive and convenient method of detecting food items. This enables users to make informed judgments and stay away from potentially hazardous foods, which can be especially helpful for those who have dietary restrictions or allergies. Additionally, the system can offer helpful details on the quality as well as the freshness of the food items, which also enables the consumers to assess their suitability before eating. This technique can also be used to highlight the cultural significance of food, enhancing culinary experiences.

The paper employs a thorough approach to deal with the challenge of food identification by collecting the labeled data of 1200 audio files for 20 distinct food items. Signal processing techniques, including spectrograms, spectral rolloff, spectral bandwidth, and mel-frequency cepstral coefficients, are applied for extracting meaningful features from the audio files. These techniques effectively capture the unique characteristics of different food items, enabling accurate classification based on their eating sounds. To learn and recognize the spectral and temporal patterns in the audio signals, various deep learning models as mentioned in Sect. “Feature extractions” are fine-tuned and trained.

**Figure 6:**
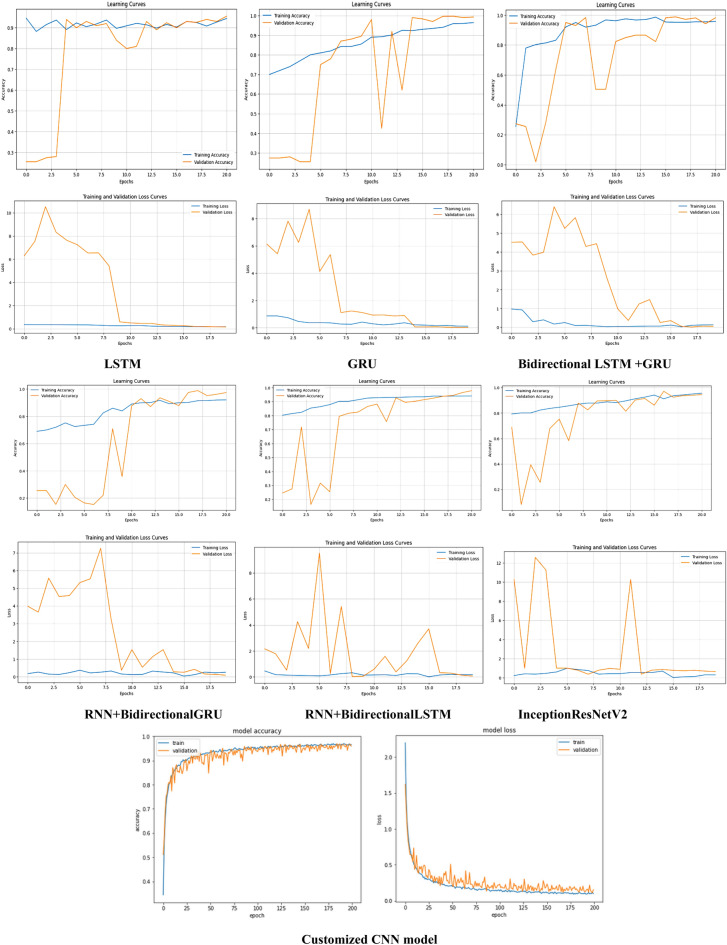
Graphical analysis of deep learning models.

These models have been evaluated using various parameters as mentioned in Sect. “Results” and their graphical curves on the basis of loss and accuracy are shown in Fig. 6. It can be seen that the good fit of learning curves in terms of accuracy and loss has been shown by customized CNN model as there is no such gap between the validation and training performances of the model irrespective of noise in them. On the contrary, the other models display certain gaps and high peaks of validation accuracy and loss which signifies that the model is overfitting the training data.

The comparison and evaluation of different deep learning models provide insights into their performance for food identification tasks. This analysis helps identify the most suitable models for accurately recognizing food items based on eating sounds. Additionally, the exploration of hybrid models, such as Bidirectional LSTM + GRU and RNN + Bidirectional LSTM, showcases the potential benefits of combining different architectures to improve classification performance.

The models' training times have also been determined in Table 11, with LSTM, a form of recurrent neural network (RNN), taking 1 h and 58 min to train. Another form of RNN, GRU, takes 5 h and 4 min of training. The training duration is reduced to 4 h and 35 min when LSTM and GRU are coupled in the LSTM + GRU model. It takes 6 h and 25 min to train the SimpleRNN + bidirectional GRU model and 9 h and 45 min to train the SimpleRNN + Bidirectional LSTM model. The deep convolutional neural network (CNN) architecture InceptionResNetV2 takes 5 h and 20 min to train. Finally, training a custom model takes 7 h.

**Table 11:** Time Frame of the applied models.

Algorithms	Time frame
LSTM	1 h 58 min
GRU	5 h 04 min
LSTM + GRU	4 h 35 min
SimpleRNN + Bidirectional GRU	6 h 25 min
SimpleRNN + Bidirectional LSTM	9 h 45 min
InceptionResNetV2	5 h 20 min
CNN custom model	7 h

These training times reflect the computational resources and they are not fixed as they rely on the configuration of the system.

However, various challenges has been recognized and handled in order for the proposed methodology to be effective and practicable. One of the most difficult tasks is assembling and curating a comprehensive dataset that includes a diverse range of food items and dining settings. Eating sound variability, such as varied eating methods, utensils, and background noise, can introduce variability that must be properly regulated in order to obtain efficient identification. Another issue is the extraction of features from audio files. While the study used spectrograms, spectral rolloff, spectral bandwidth, and mel-frequency cepstral coefficients, there may be opportunity for further research into more advanced feature extraction approaches. These approaches may be able to work on greater details and remove nuances in eating noises, resulting in better classification accuracy.

Furthermore, the proposed system's scalability and real-time implementation should be investigated. As the dataset grows and more food items are considered, it is critical to keep the computing requirements modest. Furthermore, investigating the system's deployment in practical settings such as mobile applications or embedded systems will facilitate real-time food detection, making it more accessible to a wider audience.

## Conclusion

This paper describes a novel method for food identification based on eating sounds that employs several deep learning models. The study effectively demonstrated the capability of deep learning algorithms to reliably identify food items based on their distinct sound patterns. The developed approach has a lot of potential for helping people with dietary restrictions, allergen avoidance, food quality assessment, and cultural understanding. During the conduct of this research, the data had been collected in the form of 1200 audio samples for 20 food products. Although signal processing techniques were used to extract relevant characteristics from the audio recordings, further advances in feature extraction approaches could improve the system's performance. Furthermore, selecting and fine-tuning deep learning models was a hurdle, necessitating extensive experimentation to determine the most effective architectures. Despite these obstacles, the findings of this investigation are optimistic. Deep learning methods such as LSTM, GRU, InceptionResNetV2, and a customized CNN model were used to learn and recognize spectral and temporal patterns in food-eating sounds. Besides this, the models were also hybridized such as BidirectionalLSTM + GRU and RNN + BidirectionalLSTM and were examined based on their accuracy, precision, F1 score, and recall.

The outcomes of this study suggest various possibilities for further investigation. Firstly, expanding the size of the dataset, encompassing a diverse range of food items and dining scenarios, is recommended to enhance the system's adaptability. Additionally, fine-tuning the layers of the customized CNN model and exploring more advanced deep learning architectures and approaches are crucial for boosting accuracy and robustness. Moreover, it would be beneficial to explore the real-time implementation and practical applications of the suggested methodology in real-world scenarios such as nutritional tracking apps, allergen detection systems, and culinary cultural preservation. Finally, this study shows a considerable advancement in food identification using deep learning models based on eating sounds. It demonstrates the potential of these models for accurately recognizing food products and lays the groundwork for future study in this subject. With future adjustments and study, the proposed methodology offers significant promise for numerous applications in nutrition, dietary planning, as well as food-related sectors.

## Data Availability

The dataset used in the study is openly available at the following link. https://www.kaggle.com/datasets/mashijie/eating-sound-collection.
